# Association between pulmonologists’ tobacco use and their effort in promoting smoking cessation in Turkey: a cross-sectional study

**DOI:** 10.1186/s12890-015-0131-y

**Published:** 2015-11-11

**Authors:** Pinar Pazarli Bostan, Canan Karaman Demir, Osman Elbek, Şule Akçay

**Affiliations:** School of Health, Sakarya University, Sakarya, Turkey; Occupational Diseases Education Clinic, Ankara Atatürk Chest Diseases and Thoracic Surgery Training Hospital, Ankara, Turkey; Pulmonary Medicine, Medical Faculty, Adnan Menderes University, Aydın, Turkey; Pulmonary Medicine, Medical Faculty, Başkent University, Ankara, Turkey

**Keywords:** Tobacco use, Physician, Pulmonologist

## Abstract

**Background:**

A strategy to reduce the number of smoking-related deaths is to encourage the involvement of health-care professionals in tobacco-use prevention activities and cessation counseling. Previous studies have shown that physicians’ smoking status affects their efforts to provide smoking cessation counseling. This study investigates the association between pulmonologists’ tobacco use and their efforts in promoting smoking cessation during their routine clinical practices in Turkey.

**Methods:**

This cross-sectional study was performed among active members of the Turkish Thoracic Society (TTS) between June 2010 and February 2011 using an Internet-based self-administered questionnaire. Participants gave their written informed consent. The survey included questions about responders’ sociodemographics, smoking status, and their routine clinical practice for smoking cessation counseling using the basic 5A’s (Ask, Advise, Assess, Assist, and Arrange) of smoking cessation counseling. According to the total score for the 5A’s protocol, smoking cessation counseling was dichotomized into low- and high-effort groups in promoting smoking cessation. Pearson’s chi-square test and *t*-test were used to compare groups and logistic regression models for the research question, which was approved by the TTS Scientific Ethical Committee.

**Results:**

The response rate was 41 % (*N* = 699/1701); 9.9 % were current smokers, and 72.7 % indicated that they provided high effort in promoting smoking cessation. A univariate analysis showed that noncurrent smokers were more likely to make a high effort than current smokers (odds ratio [OR], 1.82; 95 % confidence interval [CI]: 1.09–3.05; *P* = 0.02). However, there was no association between tobacco use (current smoking) and making high effort in promoting smoking cessation after controlling for the two confounders, sex and practicing in smoking cessation outpatient clinic (OR, 1.47; 95 % CI: 0.86–2.50; *P* = 0.1).

**Conclusions:**

Despite low response rate in our study and suspicions of underreporting, the smoking rate among the pulmonologists in our study was high. Non-current smokers were more likely to provide high effort in promoting smoking cessation compared to current smokers in univariate analysis. However, after controlling for the two confounders, sex and practising in SCOC, there was no association between tobacco use and providing high effort in promoting smoking cessation. Thus, improving medical school education, specialty training and post-graduate training on smoking cessation counseling may positively affect physician' effort in promoting smoking cessation.

## Background

Smoking is the most important public health problem and preventable cause of mortality in Turkey, where 27.1 % of individuals aged 15 years and older smoke daily or less than daily (41.5 % of men; 13.1 % of women) [1]. More than 100,000 people die every year as a consequence of smoking (a quarter of all deaths); this is estimated to rise to 240,000 by 2030 [2].

Physicians have a critical role in reducing tobacco use. One of the strategies to reduce the number of smoking-related deaths is to encourage the involvement of health-care professionals in the prevention of tobacco use and cessation counseling [3–5]. Smoking cessation counseling increases smoking cessation rates and encourages smokers who are not ready to quit to contemplate doing so. The Clinical Practice Guideline for Treating Tobacco Use and Dependence advocates the use of the 5A’s (Ask, Advise, Assess, Assist, and Arrange) protocol of tobacco cessation intervention to be delivered by health-care practitioners [4]. The statement of the Joint Committee on Smoking and Health suggests that clinicians should provide at least a brief intervention (first 3A’s of the 5A’s protocol) to every patient who uses tobacco [6]. Even brief and simple advice from physicians can increase smoking cessation rates substantially [4]. Clinicians have come to accept their responsibility for the first 3A’s, but are usually reluctant to provide assistance and follow up because these are time consuming and require skills that they do not have.

Although pulmonologists do not have different responsibilities in tobacco control than other physicians, they potentially have more opportunities to interact with patients who are at higher risk of tobacco-related diseases and mortality. Previous studies have shown that smokers who have chronic diseases were more likely to receive advice about quitting from providers [7–9]. A study conducted in a university hospital, which investigated physicians’ smoking cessation practices in Turkey, showed that physicians working in respiratory medicine departments were more likely to ask about patients’ smoking status and to conduct smoking cessation intervention than were physicians working in other departments [10]. In Turkey, smoking cessation counseling is mainly performed by pulmonologists, public health specialists, and family physicians. Since 2000, training for this counseling has been provided by the pulmonology societies. According to the 2011 legislation, only physicians certified by the Turkish Ministry of Health can operate smoking cessation outpatient clinics (SCOCs).

Previous studies have shown that physicians’ smoking status affects their interventions to provide smoking cessation counseling [11–13]. A collaborative study with the World Health Organization (WHO), United States Centers for Disease Control and Prevention, Turkish Ministry of Health, and the Turkish Society of Public Health Specialists found that the rate of current smokers among 4761 health-care professionals working at Turkish Ministry of Health institutions was 31 % (not limited to pulmonologists) and specialists who are smokers were less likely to provide smoking cessation counseling [14]. A study of pulmonologists who are Turkish Thoracic Society (TTS) members found that the rate of ever smoking was 31 % [15].

We conducted this study to investigate the association between pulmonologists’ tobacco use and their efforts in promoting smoking cessation during their routine clinical practice in Turkey.

## Methods

### Study design and participants

Between June 2010 and February 2011, we conducted this cross-sectional study among active members of the TTS, which is an organization for health-care professionals, mostly including pulmonologists. The results of this study were derived through reanalyzing the data from a TTS scientific research project called “Attitudes and behavior of the pulmonologist members of TTS toward smoking cessation help.” The study protocol was approved by the TTS Scientific Ethical Committee.

We used information from TTS to identify all of their active pulmonologist members, who were defined as having a current membership with a valid e-mail address. The number of active pulmonologist members during the study period was 1701. After performing a pretest, the TTS secretariat distributed an e-mail inviting these members to participate in the study about their routine smoking cessation counseling practices. The e-mail included a link to the Internet-based self-administered questionnaire, containing a written informed consent form for participation. After 30 days, a reminder was sent to improve the number of participants, and then every 15 days, regardless of whether respondents had responded previously. The reminders told recipients not to return the questionnaire if they had submitted a completed questionnaire previously. The Internet-based survey only allowed fully completed questionnaires; therefore, physicians who started but failed to complete the survey were considered as nonresponders. We had no way to monitor which physicians started the survey and failed to complete it, and no way to monitor who did not receive the e-mails. The response rate was calculated as the number of fully answered questionnaires divided by the number of active pulmonologist members of the TTS.

The survey included questions about demographics (gender, age, graduation date from medical institution, specialist or resident, academic title, and institution), smoking status of responders, and routine clinical practices using the basic items of smoking cessation counseling [16]. Any specific training on smoking cessation counseling was not performed with the study group before data collection. Only physicians who had been educated about providing smoking cessation help previously could create a difference. For this reason, the question about physician education was included in the questionnaire. In Turkey, only Ministry of Health-certified physicians can actively practice in SCOCs. Questions about this subject were also included in the questionnaire.

The survey addressed the 5A’s (Ask, Advise, Assess, Assist, and Arrange) of smoking cessation counseling [17, 18]. Each of the 5A’s protocol items were measured on a 4-point Likert scale from “never” to “always” that dichotomized responses (0 points for “never or sometimes” and 1 point for “frequently or always”). Scores were added across the five components. According to the total 5A’s protocol score, smoking cessation counseling was dichotomized into low-and high-effort groups in promoting smoking cessation, which were defined as having scores of 1–3 and ≥4, respectively.

For the analyses, the date of graduation was dichotomized into graduated before and after 1996 (the year of the first tobacco control legislation in Turkey). Physicians’ characteristics were defined as: being a specialist or resident; working in urban clinics; working in a training hospital; having an academic title; practicing in a SCOC; educated in smoking cessation help; and being a member of the Local Tobacco Control Committee (LTCC).

We used the WHO classification to define smoking status [19]. Current smokers were defined as individuals who had smoked for at least 6 months during their lifetime and were still smoking at the time of the survey. Noncurrent smokers were defined as former (ex-) smokers (smoked for at least 6 months during their lifetime, but had not smoked within the 6 months before the survey), recent quitters (smoked for at least 6 months during their lifetime, but had not smoked for less than 6 months before the survey), and never smokers (had never smoked or had smoked for fewer than 6 months or <100 cigarettes during their lifetime). We included recent quitters as noncurrent smokers—although they were not actually former smokers—because recent quitters are usually more willing to participate in interventions against smoking.

### Statistical methods

A descriptive analysis was performed for demographic features. Differences in proportion were assessed by Pearson’s chi-square test. For statistical analyses, an independent samples *t*-test was used for continuous data with normal distribution, and the Mann–Whitney *U* test was used for the data not normally distributed. Logistic regression was used to assess the association between pulmonologists’ tobacco use and their efforts in promoting smoking cessation after controlling for the potential confounders. Odds ratios (OR) and corresponding 95 % confidence intervals (95 % CI) were computed to assess the strength of associations. A *P* value <0.05 was considered statistically significant. All analyses were conducted with SPSS software for Windows (v. 13.0; SPSS Inc., Chicago, IL, USA).

## Results

The response rate was 41 % (*N* = 699/1701). Among all respondents, 9.9 % (*n* = 69) were current smokers and the rest were noncurrent smokers (never smokers, 69.1 %; former smokers, 19.5 %; recent quitters, 1.6 %). Current smokers were mostly males (current smokers, 55 %; noncurrent smokers, 32.5 %; *P* = 0.001). Noncurrent smokers were more frequently practicing in a SCOC than were current smokers (41.6 % vs 20.3 %; *P* = 0.001) (Table 1).

**Table 1:** Characteristics of current and noncurrent smokers among Turkish Thoracic Society pulmonologists

	Current smokers	Noncurrent smokers	*P* value
	*N* = 69 col%	*N* = 630 col%	
Female sex	44.9	67.5	0.001
Age (mean ± SD)	38.7 ± 7.8	39.5 ± 9.1	0.4
Pulmonology specialist	84.1	80.6	0.4
Graduated after 1996	46.4	54.9	0.1
Working in urban clinics	91.3	89.8	0.7
Working in training hospital	44.6	50.7	0.3
Academic title	18.8	27.5	0.1
Practicing in a SCOC	20.3	41.6	0.001^a^
Educated in SC help	43.5	53.3	0.1
LTCC member	7.2	9.2	0.7

*SC* smoking cessation, *SCOC* smoking cessation outpatient clinic, *SD* standard deviation, *LTCC* Local Tobacco Control Committee

^a^Continuity correction

Among all respondents, 72.7 % (70.1 % of never smokers, and 74 % of former smokers) were making high efforts in promoting smoking cessation. Compared with the low-effort group, the high-effort group had significantly higher proportions of noncurrent smokers (91.7 % vs 85.9 %), female sex (67.8 % vs 59.7 %), pulmonology specialists (84.6 % vs 71.2 %), graduation after 1996 (60.0 % vs 38.2 %), academic title (30.5 % vs 16.2 %), practicing in a SCOC (45.1 % vs 24.6 %), educated about smoking cessation help (59.3 % vs 34.0 %), and LTCC membership (11.8 % vs 1.6 %) (Table 2).

**Table 2:** Characteristics of low- versus high-effort groups promoting smoking cessation among Turkish Thoracic Society pulmonologists

	High effort in promoting SC	Low effort in promoting SC	*P* value
	*N* = 508 col%	*N* = 191 col%	
Noncurrent smoker	91.7	85.9	0.02
Female sex	67.3	59.7	0.05
Age (mean ± SD)	40.5 ± 9.1	36.4 ± 7.8	0.001
Pulmonology specialist	84.6	71.2	0.001
Graduated after 1996	60.0	38.2	0.001
Working in urban clinics	90.4	89.0	0.5
Working in training hospital	47.0	40.3	0.1
Academic title	30.5	16.2	0.001
Practicing in a SCOC	45.1	24.6	0.001
Educated in SC help	59.3	34.0	0.001
Member of LTCC	11.8	1.6	0.001

*SC* smoking cessation, *SCOC* smoking cessation outpatient clinic, *SD* standard deviation, *LTCC* Local Tobacco Control Committee

Univariate analysis shows that noncurrent smokers were more likely to make high effort in promoting smoking cessation compared with current smokers (OR, 1.82; 95 % CI: 1.09–3.05; *P* = 0.02). Sex and practicing in a SCOC were also associated with making high efforts in promoting smoking cessation (Table 2). These two variables were accepted as confounders for our research question because they were also significantly related to tobacco use (Table 1) and only these two variables were used in the logistic regression model. However, after controlling for the two confounders (sex and practicing in a SCOC), there was no association between tobacco use (current smoking) and making high effort in promoting smoking cessation (OR, 1.47; 95 % CI: 0.86–2.50; *P* = 0.1) (Table 3).

**Table 3:** Association between physicians’ tobacco use and making high efforts in promoting smoking cessation, in a multivariate analysis controlling for sex and practicing in a SCOC

	OR	95 % CI	*P* value
Noncurrent smoker	1.47	0.86–2.50	0.15
Practicing in a SCOC	2.41	1.65–3.51	<0.001
Female sex	1.30	0.91–1.85	0.14

*CI* confidence interval, *OR*, odds ratio, *SCOC* smoking cessation outpatient clinic

## Discussion

Our study showed that current smoking was associated with a low rate of making high efforts in promoting smoking cessation, which was measured by the responses to the basic five items (5A’s) of smoking cessation counseling. However, this association lost its significance when it was controlled for sex and practicing in a SCOC.

Many studies in the literature have highlighted the association between physicians’ tobacco use and providing smoking cessation help to their smoking patients [10–12]. Ohida and colleagues [20] suggested that nonsmoking physicians have more unfavorable views towards smoking and are more active in encouraging patients not to smoke. In Greece, Japan, Finland, and Estonia, smoking physicians were less likely to discuss tobacco use with patients or assist them in cessation [21].

However, the literature indicates that there are factors other than physicians’ tobacco use associated with smoking cessation intervention. Zhou and colleagues [12] found that asking patients whether they smoked and recording their smoking status in the medical record was significantly associated with being female and being very well or somewhat prepared to counsel patients about smoking cessation. A cross-sectional mail survey conducted in a random sample of general practitioners in Montreal found that the correlates of smoking cessation counseling activity were female gender, high self-efficacy, and favorable views about smoking cessation help. Correlations with offering supplementary support included awareness of stages of change in smoking cessation periods and knowledge of community resources to help patients quit [22]. In another study, physicians were significantly more likely to ask about or advise against smoking if they believed that counseling about the health hazards of smoking helped smokers to quit or if they believe that most smokers would follow smoking cessation advice [23]. All these results are similar to our findings. In the univariate analysis, we found that the rate of female sex in the high-effort group was significantly higher than the low-effort group. The reason for this sex difference is an open area for further research, but could be because of differences in smoking rates between the sexes.

Some factors (pulmonology specialist, academic title, practicing in a SCOC, educated in smoking cessation help, and LTCC member) that were related to making high efforts in promoting smoking cessation can be thought of as the determinants of high self-efficacy and favorable views about smoking cessation help. Specifically, a physician who has training and/or experience in smoking cessation counseling would be highly likely to be aware of the stages of change in smoking cessation periods. The transtheoretical model (TTM) is known by the term, “stages of change.” According to TTM, certain principles and processes of change work best at each stage to reduce resistance, facilitate progress, and prevent relapse [24]. Using TTM, a physician may provide appropriate counseling interventions tailored to the smoker’s stage of readiness to quit.

Because of the low response rate in our study, drawing inference from these findings to pulmonary physicians’ attitudes and behaviors of smoking cessation counseling is difficult [25]. Our data were based on voluntary self-reporting. Therefore, respondents in our study could have more likely included a self-selected population who are aware of the importance of smoking cessation and provided counseling. Smoking rates might be underreported while counseling could be over-reported. We do not know the smoking rates of the nonresponders. The results cannot be generalized because of this information and selection bias, and this bias could be responsible for the lack of association between physicians’ tobacco use and making high efforts in promoting smoking cessation during their routine clinical practice.

## Conclusions

On one hand, a univariate analysis shows that noncurrent smokers were more likely to make high efforts in promoting smoking cessation compared with current smokers. However, after controlling for the two confounders, sex and practicing in SCOC, there was no association between physicians’ tobacco use and making high efforts in promoting smoking cessation. Improving medical school education, specialty training, and postgraduate training on smoking cessation counseling may have a greater influence on physicians in promoting smoking cessation, regardless of their tobacco use.

On the other hand, as shown by previous studies, nonsmoker physicians are more eager to provide smoking cessation counseling. Therefore, if we want to improve physician collaboration in smoking cessation counseling programs, physicians should have priority among the groups targeted by national smoking cessation programs. Despite the low response rate in our study and suspicion of underreporting, the smoking rate among pulmonologists in our study was as high as the rate among American physicians (2.3 %) [26]. Increasing the smoking cessation rate among pulmonologists could indirectly promote their smoking cessation counseling practice. This change in perspective could help us to improve public health.
